# Investigating the differences in nutritional status between successfully weaned and unsuccessfully weaned respirator patients

**DOI:** 10.1038/s41598-023-34432-0

**Published:** 2023-05-02

**Authors:** Cheng-Yang Chiang, Chou-Chin Lan, Chin-Hsuan Yang, Yi-Cheng Hou

**Affiliations:** 1 Department of Nutrition, Taipei Tzu-Chi Hospital, Buddhist Tzu-Chi Medical Foundation, New Taipei City, Taiwan No. 289, Jianguo Rd., Xindian Dist., 23142; 2 Division of Pulmonary Medicine, Taipei Tzu-Chi Hospital, Buddhist Tzu-Chi Medical Foundation, New Taipei City, Taiwan No. 289, Jianguo Rd., Xindian Dist., 23142

**Keywords:** Health care, Nutrition

## Abstract

Long-term respirator users admitted to intensive care units need to be transferred to a respiratory care center (RCC) for weaning. It may cause malnutrition in critical care patients, which may manifest as a reduction in respiratory muscle mass, lower ventilatory capacity, and decreased respiratory tolerance. This study aimed to assess that if the patients’ nutritional status were improved, it could help RCC patients to wean from respirators. All participants were recruited from the RCC of a medical foundation in the city and Taipei Tzu Chi Hospital. The indicators include serum albumin level, respirator detachment index, maximum inspiratory pressure (PImax), rapid shallow breathing index, and body composition measurements. We recorded the length of hospital stay, mortality, and RCW (respiratory care ward) referral rate for these participants and analyzed the differences in relevant research indicators between those who were and weren’t weaned off. 43 of 62 patients were weaned from respirators, while 19 failed. The resuscitation rate was 54.8%. Patients with respirator weaning had a lower number of RCC admission days (23.1 ± 11.1 days) than respirator-dependent patients (35.6 ± 7.8 days, *P* < 0.05). The PImax of successfully weaned patients had a greater reduction (− 27.09 ± 9.7 cmH_2_O) than unsuccessful ones (− 21.4 ± 10.2 cmH_2_O, *P* < 0.05). The Acute Physiology and Chronic Health Evaluation II (APACHE II) scores of successfully weaned patients (15.8 ± 5.0) were lower than those who were not (20.4 ± 8.4, *P* < 0.05). There was no significant difference in serum albumin levels between the two groups. In the successfully weaned patients, the serum albumin concentration was increased from 2.2 ± 0.3 to 2.5 ± 0.4 mg/dL, *P* < 0.05. Improved nutritional status can help RCC patients to wean from respirators.

## Introduction

In Taiwan, nearly 200,000 individuals use artificial respirators each year. Approximately 5000 individuals must be transferred to an RCW (respiratory care ward) to continue using artificial respirators^1^. In addition, long-term use of respirators has many adverse effects on patients and causes a large burden on the family support care system. Approximately 10–15% of patients will be repositioned after their endotracheal tube is removed^2^. Failure to extubate prolongs the hospital stay, increases the risk of death and medical expenses^2^. Therefore, the medical issues of patients who are long-time users of respirators cannot be ignored.

Clinically, the “weaning index” is used as the judgment, including maximum inspiratory pressure (PImax), minute ventilation, and the frequency dependence of dynamic lung compliance, tidal volume (Vt), and rapid shallow breathing index (RSBI). Among them, the RSBI is considered one of the most reliable parameters. RSBI represents the ratio of the respiratory rate to Vt when the patient's spontaneous breathing lasts for one min under the oxygen concentration of the air (29%). PImax is the outward rebound force generated by the inhalation muscles to overcome the overall respiratory system and represents the strength of the inhalation muscles. The patient may be weaned off the respirator when the value is less than 105 bpm/L or when the PImax value is less than − 20 cmH_2_O^3^. When the patient's respirator detachment index reaches the recommended value, a respiratory therapist needs to assess the patient to determine whether it can be completely detached.

Malnutrition is a common problem among hospitalized patients^4^ which affects the structure and function of the lungs and leads to poor respiratory muscle contraction, weakened ventilation capacity, reduced respiratory endurance and respiratory mechanisms, in the end, prolonging respirator dependence time. Approximately 40–50% of intensive care units (ICU) patients have malnutrition, especially after 14 days in the ICU; it occurs in 94% of critically ill patients using respirators, indicating its seriousness in this population^5^. Arora et al.^6^ pointed out that respiratory muscle weakness caused by malnutrition mainly occurs in the diaphragm and may be related to the reduction of oxygen consumption by the mitochondria. Malnutrition also increases the infection rate, morbidity, mortality, and length of hospital stay of in-patients^7,8^.

Additionally, patients who use respirators for a long time are prone to malnutrition and exhibit weight loss and tissue depletion^9–11^. This may be due to the increased physiological and metabolic rate of severely ill patients and increased caloric requirements. The increased demand, coupled with the increased secretion of stress hormones (e.g., adrenocorticotropic hormone, growth hormone, catecholamine, cortisol, and glucagon) in the body, inhibits the action of insulin. Cells have difficulty using glucose as energy, resulting in poor utilization and increased insulin levels, which prevents the formation of ketone bodies and, therefore, affects the use of heat^8^. Malnutrition also reduces the ability of the respiratory tract and alveoli to clear bacteria, increasing the risk of complications and infection. These conditions all contribute to poor respiratory function^9,12–14^.

Caloric deficiency leads to decreased respiratory epithelial regeneration and loss of lean muscle mass, causing respiratory muscle weakness and prolonging the use of respirators. Caloric excess promotes the accumulation of body fat, making the patients overweight and hypercarbic. In addition, the occurrence of hememia increases the breathing burden and prolongs the respirator's use time^8,15^.

Council on Practice^16^ recommends assessment of "postural measurements, biochemical values, clinical tests, diet history, emotional problems, and functional assessment.” Gunen et al.^17^ reported that serum albumin and body mass index (BMI) are good predictors of inpatients' prognosis. In 2004, Ambrosino et al. showed that patients with a BMI of less than 25 kg/m^2^ are at risk of malnutrition^18^. Body composition can be measured using the following methods: hydrodensitometry, air displacement plethysmography, isotope dilution, dual-energy X-ray absorptiometry (DEXA), near-infrared interactance, anthropometry, and bioimpedance analysis (BIA). BIA is commonly used in clinical practice^18^. In the young, adult, or older adults’ populations (applicable age 20–98 years), its measurement accuracy is similar to that of Dual-energy X-ray absorptiometry (DEXA) and other methods^19,20^.

There are few related studies on muscle mass among patients in respiratory care center (RCC). This study may be the first to assess dietary calorie and protein intake and body composition, analyze blood biochemical values, and explore respiratory detachment parameter data to understand the correlation between muscle mass, location, and respirator detachment among RCC patients.

## Methods

### Research methods

This study had a prospective research design. All participants were recruited from a hospital respiratory care center (RCC) and a medical foundation in the city. All patients were over 18 years old and needed to use respirators. The exclusion criteria were a fraction of inspired oxygen > 0.6%, air leaks through chest drains, severe liver disease (Child–Pugh score C), and combined Chinese medicine and acupuncture treatment. Even if the patients with respiratory infection using antibiotic therapy, they could still be part of the study since we were like to observed the whole process of successfully weaned or not. All methods were performed in accordance with relevant guidelines and regulations. The informed consent was obtained from all participants and/or their legal guardians. All experimental protocols were approved by the Buddhist Tzu Chi Medical Foundation Taipei Tzu Chi Hospital Institutional Review Board.

### Research indicators and tools

The indicators used in this study included body position measurement, serum albumin level, respirator detachment index (weaning index), and body composition measurements.

In the body position measurement aspect, the WHO formula body calculation was used: BMI = (body weight [kg])/(height [m])^2^.

The Executive Yuan Department of Health standard for BMI was adopted (light obesity: 27 ≤ BMI < 30, moderate obesity: 30 ≤ BMI < 35, and severe obesity: BMI ≥ 35), but because the patients could not stand, body weight was measured using a bed scale (DETECTO, Missouri/USA), and height was estimated by the knee height using the formula proposed by Cheng et al.^21^. Knee height was measured with the femur and tibia perpendicular to each other and the bottom of the foot perpendicular to the tibia. The length from the joint to the sole was defined as knee height and used in the following height estimation formula:

1. Male: Length (cm) = 85.10 + 1.73 × knee height (cm)—0.11 × age (years)
2. Women: Length (cm) = 91.45 + 1.53 × knee height (cm)—0.16 × age (years).

PImax and RSBI were used as indicators for respirator detachment. PImax was measured by an inspforce (BUEHRINGER, USA), and RSBI was measured using a Haloscale Wright Respirometer (FERRARIS, Germany). Body composition was analyzed using a bio-resistance body composition analyzer (InBody S10, Biospace, Korea) to estimate the participants’ fat-free weight, body fat mass, muscle mass, mineral (bone), total body water, protein, intracellular fluid, and extracellular fluid contents. The time of the baseline and the follow-up data would be measured before we gave them the commercial formula that day and for those who still rely on the respirator while others were measured when they successfully weaned.

### Research process

Family members of patients who were interested in participating in this study could only sign up the patients for participation after they had listened to the researcher's explanation, fully understood the content of the study, and signed a consent form. When participants entered the study, and when they left RCC, the researchers collected data, including basic data, disease history, medication status, blood biochemical test data, diet data, and body composition data. The diet was commercial formula for the tube feeding products and supplied them based on each patients’ body weight. The researchers also collected information on the length of hospital stay, mortality, and RCW referral rate for these participants and analyzed the differences in relevant research indicators between those who were weaned off the respirator and those who were not.

### Statistical analysis

This study analyzed the correlations between the following indicators: serum albumin level, BMI, body composition, length of hospital stay, and respirator detachment indicators. A Kolmogorov-Smirnoff test was used for normality testing; continuous variables of normal distribution were analyzed by a Student's t-test, and continuous variables of abnormal distribution were analyzed by a Mann–Whitney U test, and non-continuous category variables were analyzed by a Fisher’s chi-square test. Regarding the difference between the pre-and post-test values, a paired t-test was used for the variables with normal distribution, and a Wilcoxon signed-rank test was used for those with an abnormal distribution. A regression analysis to investigate the associated factors. The level of significance was set at *P* < 0.05. The statistical software used in this study was version 19 of SPSS (IBM Corp., Armonk, NY, USA).

## Results

A total of 86 RCC patients were included in this study. Among them, 22 patients from respiratory care centers were discharged or transferred after signing the consent form, one patient had a cardiac pacemaker, and one had finger necrosis that precluded measurement of they were excluded. All of 62 RCC patients were included in the data analysis, of whom 43 were successfully weaned off the ventilator, 11 were transferred to RCW, and 8 were transferred to ICU or died.

### Patients’ characteristics

#### Analysis of the basic data of the patients

All 62 RCC patients were grouped according to whether they were successfully weaned. Their mean age (± standard deviation) was 73.7 ± 16.0 years, of whom 35 (56.5%) were male, and 27 (43.5%) were female (Table 1).

**Table 1:** Patients’ characteristics at baseline (I) (n = 62).

Variables	All patients (n = 62)	Successful (n = 43)	Unsuccessful (n = 19)	*p*
Age (years)^a^	73.7 ± 16.0	72.0 ± 13.6	75.1 ± 20.1	0.48
Male, n (%)^c^	35 (56.5%)	29 (46.8%)	6 (9.7%)	< 0.05*
Female, n (%)^c^	17 (43.5%)	14 (22.6%)	13 (21%)	
MICU^c^	39 (62.9%)	23 (37.1%)	16 (25.8%)	< 0.05*
SICU^c^	23 (37.1%)	20 (32.3%)	3 (4.8%)	
Height (cm)	157.0 ± 8.0	158.5 ± 7.47	153.6 ± 8.46	0.26
Body weight (kg)^a e^	57.9 ± 15.1	58.6 ± 15.4	56.2 ± 14.7	0.93
BMI (kg/m^2^)^b^	23.4 ± 5.7	23.3 ± 5.6	23.7 ± 6.0	0.58
APACHE II^a e^	20.0 ± 4.5	19.2 ± 3.9	21.7 ± 5.3	< 0.05*
MV length of day^a^	22.8 ± 12.7	17.4 ± 11.0	34.8 ± 8.0	< 0.05*
RCC day^b^	26.9 ± 11.7	23.1 ± 11.1	35.6 ± 7.8	< 0.05*

n, number; MICU, Medical Intensive Care Unit; SICU, Surgical Intensive Care Unit; RCC: respiratory care center.

^a^Statistical analyses were conducted by using Student’s t-test, b: Mann–Whitney U test, c: Chi-square test,

*Indicate a statistically significant difference (*p* < 0.05) between different groups at baseline.

Of the 62 patients, 39 (62.9%) and 23 (37.1%) were from the medical intensive care unit (MICU) and surgical intensive care unit (SICU), respectively. In the successful weaning group, 23 (37.1%) and 20 (32.3%) were from the medical intensive care unit (MICU) and surgical intensive care unit (SICU), respectively. In the unsuccessful weaning group, 16 (25.8%) and 3 (4.8%) were from the medical intensive care unit (MICU) and surgical intensive care unit (SICU), respectively (Table 1). The success rate of ventilator weaning was significantly higher in the medical intensive care unit (MICU) than in the surgical intensive care unit (SICU) (*p* < 0.05) (Table 1).

Analyzing disease severity, the mean APACHE II score for the 62 patients was 20.0 ± 4.5. The mean APACHE II score of the 43 patients in the successful weaning group was 19.2 ± 3.9 points, which was significantly lower than the mean APACHE II score of 21.7 ± 5.3 points in the 19 patients in the other group (*p* < 0.05). (Table 1).

#### Analysis of dietary intake data

The mean daily caloric and protein intake of the 62 patients were 1683.8 ± 227.0 kcal and 79.1 ± 19.5 g. Among them, the average daily calorie and protein intake of 43 patients in the successful weaning group were 1722.1 ± 240.6 kcal and 82.8 ± 20.9 g, significantly higher than the average daily intake of 19 patients in the unsuccessful weaning group. As a result, the average daily caloric intake and protein intake were 1597.4 ± 167.9 kcal and 70.8 ± 13.2 g (*p* < 0.05) (Table 2).

**Table 2:** Subjects’ characteristics (III) Dietary intake, Weaning index, Body composition (n = 62).

Variables	All patients (n = 62)	Success (n = 43)	Unsuccess (n = 19)	*p* value
Dietary intake				
Energy^b^	1683.8 ± 227.0	1722.1 ± 240.6	1597.4 ± 167.9	< 0.05*
Energy/kg^a^	30.8 ± 8.2	31.0 ± 8.2	30.2 ± 8.3	
Dietary protein^b^	79.1 ± 19.5	82.8 ± 20.9	70.8 ± 13.2	< 0.05*
Protein/kg^a^	1.4 ± 0.3	1.4 ± 0.3	1.3 ± 0.3	
Weaning index				
RSBI (bpm/L)^a e^	93.5 ± 41.3	89.8 ± 42.7	101.8 ± 37.9	0.19
PImax (cmH_2_O)^b e^	− 26.6 ± 10.9	− 27.3 ± 11.9	− 25.1 ± 8.5	0.66
Body composition				
Body fat (kg)^a e^	19.9 ± 10.5	20.3 ± 10.7	19.1 ± 9.7	0.67
FFM (kg)^b f^	37.9 ± 8.9	38.3 ± 9.6	37.1 ± 7.3	0.55
SLM (kg)^a f^	35.7 ± 8.5	36.2 ± 9.1	34.5 ± 7.0	0.47
Mineral (kg)^b f^	2.7 ± 1.0	2.6 ± 0.6	3.1 ± 1.5	
Body protein (kg^)b f^	7.1 ± 1.7	7.3 ± 1.8	6.9 ± 1.4	0.62
TBW (L)^b f^	28.1 ± 6.7	28.3 ± 7.0	27.6 ± 6.2	
ECW/TBW^b f^	0.40 ± 0.03	0.4 ± 0.02	0.4 ± 0.05	0.2
SMM (kg)^a f^	19.7 ± 5.2	19.7 ± 5.6	19.0 ± 4.2	0.64
RA (kg)^a f^	2.4 ± 1.2	2.5 ± 1.3	2.0 ± 0.7	0.33
LA (kg)^b f^	2.2 ± 0.8	2.3 ± 0.8	2.0 ± 0.7	0.32
TR (kg)^b f^	18.8 ± 4.8	19.5 ± 5.0	17.1 ± 3.8	0.13
RL (kg)^bf^	5.3 ± 2.3	5.4 ± 1.7	5.1 ± 3.4	0.082
LL (kg)^a f^	4.9 ± 1.8	5.3 ± 1.8	4.6 ± 1.7	0.16

FFM, Fat-Free Mass; SLM, Soft Lean Mass; TBW, Total body water; ECW/TBW, Extracellular water / Total body water; SMM, skeletal muscle mass; RA, right arm.; LA, left arm.; TR, trunk; RL, right leg; LL, left leg.

1Values are shown as the mean ± standard deviation, number, or percentage.

Statistical analyses were conducted by using a: Student’s t-test, b: Mann–Whitney U test.

*Indicate a statistically significant difference (*p* < 0.05) between different groups at baseline.

#### Analysis of respirator disengagement metrics

The average RSBI was 93.5 ± 41.3 bpm/L when the 62 patients entered the respiratory care center (RCC). Among them, the average RSBI of 43 patients in the successful weaning group was 89.8 ± 42.7 bpm/L when they entered the RCC; the average RSBI value of the 19 patients in the unsuccessful weaning group was 101.8 when they entered the RCC. ± 37.9 bpm/L. The results showed that although the patients in the successful weaning group entered the RCC, the average RSBI value of the patients all tended to be lower than the average RSBI value of the patients in the unsuccessful weaning group, but there was no significant statistical difference (Table 1).

Analyzing the PImax, the mean of the 62 patients entering the RCC was − 26.6 ± 10.9 cmH_2_O. Among them, the average of 43 patients in the successful weaning group was − 27.3 ± 11.9 cmH_2_O when entering the RCC; the average value of the 19 patients in the unsuccessful weaning group was − 25.1 ± 8.5 cmH_2_O when entering the RCC, There was no statistically significant difference between the two groups (Table 2).

#### Analyze body composition analysis and muscle mass distribution

In Table 3, the results of univariate Logistic regression analysis, “Transfer in” (OR = 4.64, *p* = 0.028), “Energy” (OR = 1.00, *p* = 0.046), “Protein” (OR = 1.05, *p* = 0.034), “Group of formula” (OR = 3.40, *p* = 0.039), “Sex” (OR = 4.49, *p* = 0.011), “Respirator days” (OR = 0.84, *p* < 0.001), “Albumin” (OR = 0.25, *p* = 0.018) and “Height” (OR = 1.08, *p* = 0.042) reaches the significant level in the regression coefficient (*p* < 0.05) which indicates that these independent variables are significantly associated with the likelihood of successful respirator disengagement. Therefore, multivariate Logistic regression was used as an independent variable for analysis.

**Table 3:** Logistic regression that influenced the successful evacuation of the respirator.

Variable	Univariate		Multivariate	
	OR (95%CI)	*p*	OR (95%CI)	*p*
Diagnosis				
Acute lung injuryd	Ref			
Chronic lung injuryd	0.41 (0.07–2.56)	.341		
Postoperative conditiond	2.47 (0.25–24.46)	.439		
Cardiac diseased	0.00 (0.00–NA)	.999		
Neurological diseased	3.29 (0.59–18.27)	.173		
Miscellaneous causesd	0.21 (0.02–2.65)	.226		
Transfer in				
MICU	Ref		Ref	
SICU	4.64 (1.18–18.27)	.028*	2.57 (0.21–31.37)	.460
Energy	1.00 (1.00–1.01)	.046*	1.00 (1.00–1.01)	.186
Protein	1.05 (1.00–1.09)	.034*	1.06 (0.99–1.13)	.080
BMI	0.99 (0.90–1.09)	.820		
Group of formula				
Normal group	Ref		Ref	
High-protein group	3.40 (1.06–10.87)	.039*	2.66 (0.28–25.39)	.395
Age	0.99 (0.95–1.02)	.482		
Sex				
0.female	Ref		Ref	
1.male	4.49 (1.41–14.30)	.011*	28.09 (0.56–1397.38)	.094
Respirator days	0.84 (0.76–0.92)	< .001***	0.78 (0.67–0.92)	.002**
Albumin	0.25 (0.08–0.79)	.018*	0.33 (0.04–2.67)	.298
Height	1.08 (1.00–1.16)	.042*	0.89 (0.72–1.11)	.316
Weight change	0.91 (0.80–1.03)	.139		
Protein change	1.05 (0.76–1.47)	.755		
Fat change	0.95 (0.88–1.02)	.178		
SLM change	1.01 (0.93–1.09)	.826		
FFM change	1.01 (0.95–1.09)	.674		
SMM change	1.02 (0.91–1.14)	.721		
ECW change	0.01 (0.00–116.42)	.357		
SegmentalLeanRA change	0.92 (0.61–1.36)	.664		
SegmentalLeanLA change	0.86 (0.57–1.31)	.486		
SegmentalLeanTR change	0.97 (0.89–1.07)	.546		
SegmentalLeanRL change	1.05 (0.82–1.34)	.686		
SegmentalLeanLL change	0.85 (0.61–1.20)	.361		
BodyCellMass change	1.02 (0.92–1.12)	.723		
BoneMineralContents	1.48 (0.75–2.93)	.259		
APACHE score change	0.95 (0.87–1.05)	.346		
RSBI change	1.00 (0.98–1.01)	.460		
PImax change	0.99 (0.94–1.04)	.555		
Albumin value change	2.64 (0.60–11.56)	.197		
Group of albumin				
Without albumin	Ref			
With albumin	0.66 (0.22–1.96)	.453		

MICU, Medical Intensive Care Unit; SICU, Surgical Intensive Care Unit; RCC: respiratory care center. SLM, Soft Lean Mass; FFM, Fat-Free Mass; SMM, skeletal muscle mass; ECW, Extracellular water; RA, right arm.; LA, left arm.; TR, trunk; RL, right leg; LL, left leg.

The results of final multivariate Logistic regression analysis showed that only the regression coefficient of “Respirator days” (OR = 0.78, *p* = 0.002) reached a significant level (*p* < 0.05), and its OR value was lower than 1, indicating that the case the more days the respirator is used, the lower the probability of successful disengagement of the respirator; in addition, the significance of some variables is quite close to 0.05, including “protein” (OR = 1.06, *p* = 0.080). Its OR value is higher than 1, which means that the higher the protein intake of the case, the higher the probability of successful escape from the respirator.

#### Analysis of patient's blood serum albumin

The mean serum albumin concentration in the 62 patients was 2.3 ± 0.3 g/dL. Among them, the mean serum albumin concentration of 43 patients in the successful weaning group was 2.2 ± 0.3 g/dL, which was significantly lower than the mean serum albumin concentration of the 19 patients in the unsuccessful weaning group of 2.4 ± 0.3 g/dL (*p* < 0.05) (Table 3).

#### Analysis of intravenous serum albumin supplementation

Of the 62 patients, 25 (40.3%) had been supplemented with serum albumin by intravenous injection, and 37 (59.7%) had not been supplemented with serum albumin by intravenous injection. Among them, 13 (21%) of the 43 patients in the successful weaning group had supplemented serum albumin by intravenous injection, and 30 patients (48.4%) had not supplemented serum albumin by intravenous injection. Of the 19 patients in the unsuccessful weaning group, 12 (19.4%) had been supplemented with serum albumin by intravenous injection, and 7 (11.3%) had not been supplemented with serum albumin by intravenous injection. The results showed that in the successful weaning group, significantly more patients did not receive serum albumin supplementation by intravenous injection (*p* < 0.05) (Table 3).

## Discussion

### Calorie and protein intake and the patient's respirator detachment

Insufficient nutrition will lead to muscle loss, and excessive nutrition will increase the need for ventilation. Both lead to weakness of the respiratory muscles, resulting in respiratory failure and failure of respirator weaning. Therefore, providing proper nutrition for patients who use respirators for a long time can help maintain good nutritional status and aid in weaning from respirators.

According to the results of this study, the successful weaning group had a higher average daily calorie and protein intake (successful weaning group: 1722.1 ± 240.6 kcal and 82.8 ± 20.9 g, unsuccessful weaning group: 1597.4 ± 167.9 kcal and 70.8 ± 13.2 g, respectively; *P* < 0.05) (Table 3).

Cerra et al.^22^ believed that when a patient is in a chronic severe disease stage, the target calorie intake should be 18–25 kcal/kgBW/day. Doley et al.^23^ suggested that a chronically severely ill patient on prolonged respirator use should be given at least 20–30 kcal/kgBW/day. The ASPEN guidelines suggest that when there is no means of performing "indirect calorimetry," the calorie needs of critically ill patients can be calculated using commonly used evaluation formulas or should be 25–30 kcal/kgBW/d; the recommended amount of protein is 1.2–2.0 g/kgBW/d. Therefore, all participants in this study met the recommended daily calorie intake standards.

In terms of protein intake, the average amount of protein per kilogram of body weight given to the successful weaning group was 1.4 ± 0.3 g/kg, which is close to that reported by Lin et al. in their study^24^. However, according to the 24-h urine urea nitrogen (UUN) test findings and clinical conditions, it was adjusted to 1.57 ± 0.4 g/kg.

Lin et al.^24^ studied the effects of factors such as protein metabolism and nutritional status on respirator detachment in 156 patients in an RCC. Most patients were elderly individuals over 70 years (57.1%), and 42.7% were men. The results showed that before a patient was transferred to an RCC, his dietary protein intake was 1.135 ± 0.33 g/kg/day. It was increased to 1.57 ± 0.422 g/kg/day during the study period based on UUN test findings and clinical conditions. The balance increased from − 3.552 ± 4.4 to − 0.4485 ± 3.62, and RSBI decreased from 73.73 ± 59.54 to 49.5 ± 61.15. In this study the results of the successful escape from the respirator group before entering RCC and leaving RCC were compared, and the RSBI was significantly reduced (*P* < 0.05), similar to the results of Lin et al.^24^.

### Muscle consumption of patients using respirators

Breathing depends on the strength of respiratory muscles. The respiratory muscles include the diaphragm, internal intercostal muscles, external intercostal muscles, scalene muscles, and sternocleidomastoid muscles. In chronic obstructive pulmonary disease studies, the diaphragm was confirmed that the strength of respiratory muscles (PImax and PEmax) is closely related to body weight and receptor tissue (lean body mass)^25^.

In this study, the average muscle mass distribution in the right arm (RA) was 2.5 ± 1.3 kg; left arm (LA), 2.3 ± 0.8 kg; trunk (TR), 19.5 ± 5.0 kg; right leg (RL), 5.4 ± 1.7 kg; and left leg (LL), 5.3 ± 1.8 kg, and when leaving the RCC, RA was 2.3 ± 1.3 kg; LA, 2.3 ± 1.3 kg; TR, 19.0 ± 6.5 kg; RL, 5.1 ± 1.3 kg; and LL, 5.0 ± 1.7 kg. The results showed that the average muscle mass distribution of RL and LL decreased during the training process of the successfully detached from the respirator group, but this was not significant, while those of RA, LA, and TR decreased significantly (*P* < 0.05) (Table 4).

**Table 4:** Analysis of patients from respirator success and unsuccessful factors (II).

Variables	Success (n = 43)		Δ	Unsuccess (n = 19)		Δ
	Baseline	Follow-up		Baseline	Follow-up	
Body composition						
Body fat (kg)^a e^	20.3 ± 10.7	19.9 ± 11.3	− 0.4	19.1 ± 9.7	20.8 ± 12.2	1.7
FFM (kg)^b f^	38.3 ± 9.6	36.8 ± 8.7	− 1.5	37.1 ± 7.3	34.7 ± 9.1	− 2.4
SLM (kg)^a f^	36.2 ± 9.1	34.4 ± 8.4	− 1.8	34.5 ± 7.0	32.6 ± 8.6	− 1.9
Mineral (kg)^b f^	2.6 ± 0.6	2.6 ± 0.7	0	3.1 ± 1.5	2.6 ± 0.8	− 0.5
Body protein (kg^)b f^	7.3 ± 1.8	7.0 ± 1.8	− 0.3	6.9 ± 1.4	6.5 ± 1.8	− 0.4
TBW (L)^b f^	28.3 ± 7.0^+^	27.0 ± 6.4^+^	− 1.3	27.6 ± 6.2	26.2 ± 7.0	− 1.4
ECW/TBW^b f^	0.4 ± 0.02	0.4 ± 0.03	0	0.4 ± 0.05	0.4 ± 0.06	0
SMM (kg)^a f^	19.7 ± 5.6	19.1 ± 5.3	− 0.6	19.0 ± 4.2	17.6 ± 5.6	− 1.4
RA (kg)^a f^	2.5 ± 1.3^+^	2.3 ± 1.3^+^	− 0.2	2.0 ± 0.7	2.0 ± 0.7	0
LA (kg)^b f^	2.3 ± 0.8^+^	2.3 ± 1.3^+^	0	2.0 ± 0.7	2.2 ± 1.0	0.2
TR (kg)^b f^	19.5 ± 5.0^+^	19.0 ± 6.5^+^	− 0.5	17.1 ± 3.8	17.4 ± 3.9	0.3
RL (kg)^bf^	5.4 ± 1.7	5.1 ± 1.3	− 0.3	5.1 ± 3.4	4.6 ± 1.5	− 0.5
LL (kg)^a f^	5.3 ± 1.8	5.0 ± 1.7	− 0.3	4.6 ± 1.7	4.7 ± 2.0	0.1

FFM, Fat-Free Mass; SLM, Soft Lean Mass; TBW, Total body water; ECW/TBW, Extracellular water/Total body water; SMM, skeletal muscle mass; RA, right arm.; LA, left arm.; TR, trunk; RL, right leg; LL, left leg.

1Values are shown as the mean ± standard deviation, number, or percentage.

^a^Statistical analyses were conducted by using Student’s t-test, b: Mann–Whitney U test, e: paired t-test, f: Wilcoxon signed rank test.

*Indicate a statistically significant difference (*p* < 0.05) between different groups at baseline.

**Indicate a statistically significant difference (*p* < 0.05) between different groups at follow-up.

+ Indicate a statistically significant difference (*p* < 0.05) between baseline and follow-up in the success group.

+ + Indicate a statistically significant difference (*p* < 0.05) between baseline and follow-up in the unsuccess group.

The results showed that the weight loss and muscle loss of the successfully detached from the respirator group were mainly concentrated in the TR and upper limb muscles. In contrast, the unsuccessful weaning group had no weight or muscle loss. It is speculated that it may be the same as the unsuccessful weaning group during breathing training. It is related to the interruption of breathing training and rest more often.

### Malnutrition and weight change in severely ill patients

Wu et al.^26^ found that failure of respiratory detachment was related to a lower serum albumin level (albumin level < 2.6 g/dL, odds ratio = 5.1; 95% confidence interval = 1.04–24.66).

In the successful weaning group, RSBI, which was a respirator detachment index, was 89.8 ± 42.7 bpm/L and decreased to 71.1 ± 40.8 bpm/L (*P* < 0.05), and the PImax value did not change between entering and leaving the RCC, indicating that the tidal volume increased, the respiratory rate decreased, and the inspiratory force was maintained; therefore, the participants had less laborious breathing (Table 2). In addition, during training without breathing apparatus, weight loss (*P* < 0.05) and muscle loss (*P* < 0.05) were both significant, and the area of muscle loss was mainly concentrated in the breathing muscles, including TR and upper limbs (RA, LA); therefore, during the off respirator training period, the patient still needed enough calories and protein. Finally, when the participants in this group left the RCC, the serum albumin concentration of patients increased significantly compared to that before entering the RCC (pre: 2.2 ± 0.3 g/dL, back: 2.5 ± 0.4 g/dL; *P* < 0.05), and the total water content was significantly reduced compared to that before (before: 28.3 ± 7.0 kg, after: 27.0 ± 6.4 kg; *p* < 0.05), indicating that the participants’ metabolism gradually recovered. This result is similar to the systematic and integrated analysis conducted by Stieff et al.^27^.

In the unsuccessful weaning group, there were no significant differences in body weight, respirator detachment parameters, and muscle mass distribution between entering and leaving RCC. However, in this group, the number of patients who were supplemented with serum albumin by intravenous injection was higher than those who did not require supplementation (Table 1). This result may be related to the disease and edema treatment. Therefore, the number of days of respirator use and RCC hospitalization was longer.

### Factors that affect patient weaning from a respirator

The results showed that the factors related to detachment from the respirator included RCC stay in days, fewer days successfully detached from a respirator (successful detachment from the respirator group: 23.1 ± 11.1 days, unsuccessful weaning group: 35.6 ± 7.8 days; *P* < 0.05); PImax, successful weaning group was higher (successful weaning group: 27.1 ± 9.7 cmH_2_O, unsuccessful weaning group: 21.4 ± 10.2 cmH_2_O; *P* < 0.05); and disease severity APACHE II score, successful weaning group was lower (successful weaning group: 15.8 ± 5.0 points, unsuccessful weaning group: 20.4 ± 8.4 points; *P* < 0.05). These results are consistent with the results reported by Wu et al.^28^. In addition, the unsuccessful weaning group had longer RCC days, lower PImax, and APACHE II scores for disease severity than did the unsuccessful weaning group.

In this study, there was no significant difference in the serum albumin concentration between the groups. However, the serum albumin concentration increased significantly in the successful weaning group (*P* < 0.05) (Table 5). This result is similar to that reported by Wu et al.^28^. The serum albumin concentration can be used to indicate a detachment from a respirator.

**Table 5:** Analysis of patients from respirator success and unsuccessful factors.

Variables	Successful (n = 43)		Δ	Unsuccessful (n = 19)		Δ
	Baseline	Follow-up		Baseline	follow-up	
Body weight (kg)^a e^	58.6 ± 15.4^+^	56.0 ± 14.3^+^	− 2.6	56.2 ± 14.7	55.6 ± 13.0	− 0.6
BMI (kg/m^2^)^b^	23.3 ± 5.6	22.5 ± 5.4	− 0.8	23.7 ± 6.0	23.1 ± 5.5	− 0.6
APACHE II^a e^	19.2 ± 3.9*^+^	15.8 ± 5.0**^+^	− 3.4	21.7 ± 5.3*	20.4 ± 8.4**	− 1.3
Biochemical values						
Albumin (g/dL)^a e^	2.2 ± 0.3*^+^	2.5 ± 0.4^+^	0.3	2.4 ± 0.3*	2.5 ± 0.4	0.1
Weaning index						
RSBI (bpm/L) ^a e^	89.8 ± 42.7^+^	71.1 ± 40.8^+^	− 18.7	101.8 ± 37.9	81.1 ± 48.5	− 20.7
PImax (cmH_2_O)^b e^	− 27.3 ± 11.9	− 27.1 ± 9.7**	0.2	− 25.1 ± 8.5	− 21.4 ± 10.2**	3.7

n, number; BMI, body mass index; APACHE IIscore, Acute Physiology, and Chronic Health Evaluation score; RCC: respiratory care center.

^a^Statistical analyses were conducted by using Student’s t-test.

^b^Mann–Whitney U test.

^c^Chi-square test.

^d^Fisher Chi-square test.

^e^Paired t-test.

^f^Wilcoxon signed rank test.

*Indicate a statistically significant difference (*p* < 0.05) between different groups at baseline.

**Indicate a statistically significant difference (*p* < 0.05) between different groups at follow-up.

+Indicate a statistically significant difference (*p* < 0.05) between baseline and follow-up in the success group.

+ +Indicate a statistically significant difference (*p* < 0.05) between baseline and follow-up in the unsuccess group.

## Limitations, and future research directions

These data were measured by different technicians, and the measurement consistency between the technicians is unclear. Therefore, future studies can use similar research team methods to strengthen the research. In this study, anthropometrics and BIA were used. This method is based on the resistance of human adipose tissue, which may be interfered by fluid and muscle even though it is really small. The difference in resistance was used to estimate the body fat. The ratio applies to those aged 20–98 years, and its accuracy is similar to those of DEXA and other methods^19,20^.

Though the albumin is the most common value to reflect the nutritional status in clinical, it could be low when systemic inflammation happened. Inflammation and malnutrition both reduce albumin concentration by decreasing its rate of synthesis so it usually should not be used as a nutritional marker after supplementation in hospitalized patients.

During respirator disengagement, muscle consumption is concentrated in the breathing muscles (upper limbs and TR). It is recommended that more rigorous longitudinal continuity be used in the future. Interventional experimental research to continuously track changes in dietary intake, blood biochemical values, muscle mass, and muscle function of RCC patients as indicators of causality determination are warranted to improve respiratory muscle functions and effectiveness of ventilator detachment. It is recommended that future studies increase the sample size in a multi-center setup to increase the generalizability of research results.

## Conclusion

This study assessed the difference between patients who were weaned off from respirators and those who remained dependent on respirators. We found that RSBI was lower, and serum albumin concentration was higher in the successful weaning group. Additionally, weight loss and changes in body muscle composition during training were more profound in the upper body and torso-related breathing muscles than in others. Finally, these results show that improved nutritional status aids in weaning RCC patients from respirators.

## Data Availability

The data that used and/or analyzed during the current study are available from the corresponding author on reasonable request.
